# Diagnostic protocol for gestational diabetes mellitus (GDM) (IADPSG/ADA, 2011): influence on the occurrence of GDM and mild gestational hyperglycemia (MGH) and on the perinatal outcomes

**DOI:** 10.1186/s13098-016-0200-2

**Published:** 2017-01-03

**Authors:** Mariana Pinto Sirimarco, Helena Maciel Guerra, Eduardo Guimarães Lisboa, Joice Monalisa Vernini, Bianca Nicolosi Cassetari, Roberto Antonio de Araujo Costa, Marilza Vieira Cunha Rudge, Iracema de Mattos Paranhos Calderon

**Affiliations:** 1 GP Program in Gynecology, Obstetrics and Mastology (PGGOM), Botucatu Medical School/Unesp (FMB/Unesp), Botucatu, Brazil; 2 Department of Gynecology and Obstetrics, FMB/Unesp, Botucatu, Brazil

**Keywords:** Gestational diabetes mellitus, Mild gestational hyperglycemia, Oral glucose tolerance test, Diagnosis, Perinatal outcomes

## Abstract

**Background:**

In August 2011, the Specialized Center for Diabetes and Pregnancy of the Botucatu Medical School/Unesp adopted a new diagnostic protocol for gestational diabetes mellitus, recommended by the American Diabetes Association and the International Association of the Diabetes and Pregnancy Study Group. The glycemic profile was evaluated using the 75-g oral glucose tolerance test (OGTT) used to diagnose mild gestational hyperglycemia, recognized and treated in our department as gestational diabetes mellitus. The cost-effectiveness of the new guidelines and the continued need for the evaluation of the glycemic profile, as part of our Service protocol, are controversial and require further investigation. We aimed to assess the impact of the new guidelines on the evaluation of mild gestational hyperglycemia and gestational diabetes mellitus, the incidence of adverse perinatal outcomes, and the association between the 75-g OGTT and the glycemic profile for the diagnosis of mild gestational hyperglycemia.

**Methods:**

This cross-sectional study was performed identifying a convenience sample of pregnant women and their newborns. The women used our Service for diagnostic procedures, prenatal care and delivery, both before (January 2008 to August 14, 2011) and after (August 15, 2011 to December 2014) the protocol modification. The following variables were compared, following stratification according to diagnostic protocol: prevalence of gestational diabetes mellitus and mild gestational hyperglycemia, newborns large for gestational age, macrosomia, first cesarean delivery, and newborn hospital stay. Statistical analysis was performed using Poisson regression, the Student’s t test, the Chi square or Fisher’s exact test and risk estimate. The statistical significance threshold was set at 95% (p < 0.05).

**Results:**

The new protocol resulted in an 85% increase in the number of women with GDM, but failed to identify 17.3% of pregnant women classified as having mild gestational hyperglycemia, despite a normal 75-g OGTT. The new guidelines did not affect perinatal outcome.

**Conclusions:**

These results support the validity of maintaining the glycemic profile as part of the diagnostic protocol at our hospital. Large multicenter studies with an adequate sample size are required for conclusive evidence on the cost-effectiveness of the new protocol.

## Background

Gestational diabetes mellitus (GDM) is defined as any degree of glucose intolerance developing or first detected during pregnancy [1, 2]. Recently, the American Diabetes Association (ADA) re-defined GDM as follows: “*diabetes diagnosed in the second and third trimesters of pregnancy*” [3].

Irrespective of the definition of glucose intolerance and diabetes and regardless of the pregnancy period, this condition of hyperglycemia, if untreated, can lead to adverse perinatal outcomes (APNO). The most frequent adverse events include the increased risk of birth trauma and the higher incidence of cesarean sections, macrosomia, episodes of neonatal hypoglycemia, and respiratory distress syndrome and/or prematurity, all of which increase the risk of perinatal death [4]. Children of mothers with GDM have a higher risk of developing obesity and metabolic syndrome (MS), which have repercussions in adulthood [4–6]. Mothers with GDM are also at an increased risk of developing type 2 diabetes mellitus (DM2) and MS in later life, as well as preeclampsia in subsequent pregnancies [7, 8].

The importance of maternal hyperglycemia, regardless of the diagnostic criteria for GDM, was first highlighted by Rudge and colleagues in 1990. The authors combined the 100-g oral glucose tolerance test (OGTT) with the glucose profile (GP) for the diagnosis of GDM. Four groups were identified on the basis of the response to these two tests: IA, IB, IIA, and IIB. In group IA, both the OGTT and GP were normal; in group IB, the OGTT was normal and the GP was changed; in group IIA, the OGTT was changed and GP was normal; and in group IIB, both test results were changed [9].

GP consisted of daytime assessments (8–6 p.m.) of maternal plasma glucose every 2 h, with a 2840 kcal diet, divided into five meals: breakfast, lunch, dinner, and two daytime snacks. Fasting glucose levels of 90 mg/dL and postprandial levels of 130 mg/dL were set as the normal reference values. Values greater than or equal to the reference values identified a changed test result, and hyperglycemia was confirmed, regardless of the outcome of the 100-g OGTT [9].

This classification and the treatment protocol are currently used in the Specialized Center for Diabetes and Pregnancy of the Clinical Hospital of Botucatu Medical School/Unesp (SEDG-FMB/Unesp). Pregnant women in group IA are followed-up in the low risk prenatal group; those in group IB with mild gestational hyperglycemia (MGH) are treated as having GDM. The pregnant women in groups IIA and IIB have GDM and receive individualized treatment, which includes exercise and dietary advice, encouragement for physical activity, and the administration of insulin, if required [9, 10].

The following findings were also observed the study by Rudge et al. Firstly, there was a comparable rate of newborns (NB) large for gestational age (LGA) born to mothers in groups IB, IIA and IIB. Secondly, the incidence of type 2 diabetes (DM2) in groups IB and IIA, 8–12 years after the index pregnancy, was equivalent. Thirdly, perinatal mortality in group IB was 10 times greater than that in group IA and comparable to that observed in the group of mothers with GDM. Furthermore, the study also identified approximately 20% of pregnant women with altered diagnostic test results requiring treatment for the control of hyperglycemia [10]. These findings highlight the importance of tracking, diagnosing and treating MGH in the same manner as GDM.

In 2011, the American Diabetes Association (ADA) recommended comprehensive changes to the diagnostic criteria for GDM [1]. A new protocol was developed by the *International Association of Diabetes and Pregnancy Study Group* (*IADPSG*) [11] on the basis of the results of the *Hyperglycemia and Adverse Pregnancy Outcome* (*HAPO*) *Study* [12]. This study included 23,316 women undergoing the 75-g OGTT between 24 and 32 weeks of pregnancy. It showed a directly proportional correlation between maternal blood glucose levels and the occurrence of predefined primary outcomes: birth weight above the 90th percentile (P90), need for a first cesarean section, neonatal hypoglycemia, and high levels of C-peptide in the umbilical cord [12].

The ADA/IADPSG diagnostic protocol [1] recommends the following: (1) investigation during the first trimester of pregnancy to identify women with overt, undiagnosed DM, by testing fasting glucose (≥126 mg/dL), glycated hemoglobin (HbA1c) (≥6.5%) or random blood glucose (≥200 mg/dL). A single confirmed changed test result is sufficient for the diagnosis of overt diabetes; (2) universal screening of all pregnant women, with no pre-existing diagnosis of overt diabetes, between 24 and 28 weeks of pregnancy. Screening consists of the 75-g OGTT and the collection of three glucose samples (fasting, 1 and 2 h after the glucose overload, respectively) where the normal limits are, respectively, 92, 180, and 153 mg/dL. One changed value is sufficient for the diagnosis of GDM [11]. This protocol was recommended by the ADA in January 2011 and implemented within our department (as a Service diagnostic protocol) from August 15, 2011. However, GP was maintained, irrespective of the outcome of the 75-g OGTT.

Several studies have assessed the impact of these new criteria on the prevalence of GDM and perinatal outcomes as well as have determined the cost-effectiveness. So far, results suggest an increased prevalence of GDM, with values ranging from 10 to 25%, and minimal effect on perinatal outcomes; however, there are significant inconsistencies. Further research is required to assess the cost-effectiveness of these new recommendations [13–17].

Following a critical review, the World Health Organization (WHO) also proposed changes to the diagnostic protocol of maternal hyperglycemia, differentiating diabetes mellitus in pregnancy (DM during pregnancy) from GDM. According to the revised WHO guidelines, irrespective of gestational age, the diagnosis of DM during pregnancy is made on the basis of fasting glucose ≥126 mg/dL; glucose ≥200 mg/dL, measured 2 h after a 75-g glucose load; or a random blood glucose level ≥200 mg/dL, associated with clinical symptoms. Fasting glucose values of 92–125 mg/dL or glucose levels of 153–199 mg/dL, measured 2 h after a 75-g glucose load, were used to confirm the diagnosis of GDM [18].

Further research is necessary to develop a single protocol, which is preferable. The combination of two diagnostic tests (OGTT and GP), as proposed by Rudge et al. [9], identifies approximately 20% of pregnant women with altered test results requiring treatment to control hyperglycemia [10]. This is comparable to the prevalence of GDM identified under the new ADA/IADPSG diagnostic criteria [11]. This conclusion raises doubts about the necessity of maintaining the GP as part of the Service diagnostic protocol because, with the reduced and more comprehensive limits of the 75-g OGTT, patients with MGH could now meet the diagnostic criteria of GDM.

The validity of the changes proposed by the new ADA/IADPSG protocol is controversial [1, 11], yet the new protocol is already established as part of the Service since August 2011. In this context, the proposal for the present study is justified. It is anticipated that the research will identify changes in the prevalence of GDM, define the role of GP in the MGH diagnostic protocol, evaluate the occurrence of APNO in pregnancies complicated by hyperglycemia or diabetes and, above all, contribute to improvements in the quality of the Service provided by our unit.

The aim of this study was to evaluate the impact of the new ADA/IADPSG protocol [1] on the prevalence of MGH and GDM, on the occurrence of APNO, and on the combination of the 75-g OGTT and GP for the diagnosis of MGH in SEDG-FMB/Unesp.

## Methods

### Design and study site

This cross-sectional cohort study was performed analyzing data stored in the SEDG/FMB-Unesp database. A convenience sample was defined; this included pregnant women who underwent diagnostic tests, prenatal care, and delivery, as part of the Service, before (January 2008 to August 14, 2011) and after (August 15, 2011 to December 2014) the revisions to the GDM diagnostic protocol. The women’s newborns were also included in the study.

### Study groups

Before the adoption of the new criteria, the Service diagnostic protocol included the 100-g OGTT, as set out by the 2010 ADA guidelines [19], combined with the GP [9, 10]. Following the publication of the 2011 ADA guidelines for GDM, the Service adopted a new diagnostic protocol, replacing the 100-g OGTT with the 75-g OGTT, while maintaining the combined approach with the glycemic profile [9, 10].

Therefore, both before and after the introduction of the new criteria, and despite the changes in the glucose load administered as part of the OGTT, the Service has always recognized Rudge’s classification: IA (normal OGTT and GP), IB (normal OGTT and changed GP—MGH), IIA (changed OGTT and normal GP—GDM), and IIB (changed OGTT and GP (GDM) [9]). For the purposes of this study, no distinction was made between Rudge’s groups IIA and IIB. Therefore, only three groups of pregnant women were identified:

- Nondiabetic (ND—control).
- Mild gestational hyperglycemia (MGH—normal OGTT and changed GP).
- Gestational diabetes mellitus (GDM).

We compared the prevalence and perinatal outcomes of these groups, in predefined periods before and after the implementation of protocol changes (August 15, 2011).

### Perinatal outcomes

NB-LGA, defined as weight for gestational age ≥P90 [10]: number (n) and percentage (%);

- Macrosomia, defined as birth weight ≥4000 g [10]: number (n) and percentage (%);
- First C-section, as an indirect marker of fetal macrosomia [12]: number (n) and percentage (%);
- Length of hospital stay, as an indirect marker of NB morbidity [10], defined by the time between birth and discharge of the NB, categorized as up to 3 days, 4–7 days, and over 7 days.

### Treatment protocol

All pregnant patients diagnosed with MGH and GDM underwent the same treatment under the Service protocol both before and after the introduction of protocol changes [10]. This protocol includes guidance on adequate nutrition from nutritionists, encouragement to exercise regularly and, whenever necessary, insulin associated with dietary advice and exercise [20]. The control of maternal hyperglycemia was assessed by the glycemic profile at least every 15 days. When the average glycemia was ≥120 mg/dL (calculated as the arithmetic mean of all blood glucose levels, measured as part of the GP), NPH insulin was administered with doses and application times adjusted according to the glucose values and hyperglycemic peaks observed [10].

### Statistical analysis

The relevant data were extracted from the database of the Service and saved in Excel 2010 spreadsheets. Data were checked for consistency of information.

We identified the relevant time periods for analysis, the diagnostic protocols (defined as “OLD” and “NEW”) and the resulting diagnoses, and the APNO. The mean values and their respective standard deviations were compared using Poisson regression and the Student’s t test. The Chi square or Fisher’s exact test were used to evaluate the associations between variables. We calculated the relative risk (RR) and the 95% confidence interval for APNO according to diagnostic protocols. A p value of <0.05 was considered statistically significant.

## Results

There were no significant differences in the ND, MGH, or GDM groups with stratification of the subjects according to the OLD and NEW protocols. The only exception was the average number of previous cesareans in the GDM group, which was lower in women under the NEW protocol (*p* = 0.0370) (Table 1). The blood glucose levels at 1 and 2 h post-glucose load were lower under the NEW protocol across groups (ND, MGH and GDM). Fasting glucose was lower in MGH pregnant women under the NEW protocol than under the OLD protocol. GDM maintained during pregnancy was lower in the group of GDM pregnant women under the NEW protocol. The HbA1c level at the end of pregnancy was lower in ND pregnant women identified by the NEW protocol while no significant differences were observed in the MGH and GDM groups regardless of the diagnostic protocol used (Table 2).

**Table 1:** Population characteristics in the ND, MGH and GDM groups stratified according to diagnostic protocol

	ND (N = 199)			MGH (N = 89)			GDM (N = 194)		
	OLD	NEW	*p*	OLD	NEW	*p*	OLD	NEW	*p*
Pregnancies	2.59 ± 1.54	2.27 ± 1.54	0.1672	3.27 ± 2.05	3.14 ± 1.90	0.7368	3.30 ± 1.56	3.15 ± 1.58	0.5850
Previous C-sections	0.46 ± 0.77	0.40 ± 0.74	0.5094	1.03 ± 1.14	0.62 ± 0.83	*0.0370*	0.83 ± 0.87	0.64 ± 0.71	0.1596
Initial BMI (kg/m^2^)	28.83 ± 7.79	26.75 ± 5.85	0.1450	32.86 ± 4.29	30.38 ± 8.47	0.3976	31.13 ± 4.84	32.50 ± 7.28	0.4379
Final BMI (kg/m^2^)	33.40 ± 8.25	31.62 ± 5.27	0.3296	37.37 ± 4.89	34.36 ± 7.99	0.2579	35.01 ± 4.24	36.50 ± 6.30	0.3336
WG (kg)	15.11 ± 10.90	12.16 ± 6.80	0.1736	12.10 ± 6.65	10.52 ± 5.60	0.4356	10.69 ± 8.32	9.87 ± 7.84	0.6444

Results expressed as mean ± SD

Poisson regression for the number of pregnancies and previous C-section

Student’s t test for the other analyses

*ND* non diabetic, *MGH* mild gestational hyperglycemia, *GDM* gestational diabetes mellitus, *WG* weight gain

**Table 2:** Results of the diagnostic tests and maternal glycemic control in the ND, MGH, and GDM groups stratified according to diagnostic protocol

	ND (N = 199)			MGH (N = 89)			GDM (N = 194)		
	OLD	NEW	*p*	OLD	NEW	*p*	OLD	NEW	*p*
GTT- fasting (mg/dL)	74.17 ± 7.22	72.54 ± 6.39	0.0979	84.13 ± 9.24	79.06 ± 7.97	*0.0071*	97.46 ± 21.74	93.41 ± 11.53	0.1937
GTT-1 h (mg/dL)	124.90 ± 29.91	109.00 ± 25.06	*<0.0001*	144.40 ± 23.45	135.30 ± 25.15	0.0864	197.40 ± 38.60	174.10 ± 33.70	*0.0001*
GTT-2 h (mg/dL)	102.70 ± 23.76	90.92 ± 19.60	*0.0002*	128.80 ± 24.42	114.90 ± 21.42	*0.0057*	191.40 ± 47.06	148.8- ± 33.54	**<** *0.0001*
GTT-3 h (mg/dL)	92.66 ± 20.79	–	–	110.53 ± 24.88	–	–	145.00 ± 41.97	–	
GA (mg/dL)	83.35 ± 10.29	82.14 ± 7.40	0.3397	97.16 ± 7.49	95.31 ± 6.14	0.2056	106.30 ± 10.28	99.95 ± 8.83	**<** *0.0001*
HbA1c/delivery (%)	5.43 ± 0.40	5.17 ± 0.45	*0.0055*	5.57 ± 0.53	5.43 ± 0.54	0.3855	5.81 ± 0.71	5.64 ± 0.63	0.2516

Results expressed as mean ± SD

Student’s t test for all analyzes

*GA* Glycemic average maintained during pregnancy, *HbA1c* glycated hemoglobin, *ND* non diabetic, *MGH* mild gestational hyperglycemia, *GDM* gestational diabetes mellitus

The NEW protocol did not influence the prevalence of pregnant women in the ND, MGH, or GDM groups. Nevertheless, 17.3% of the pregnant women under this protocol were diagnosed with MGH and did not satisfy the new diagnostic criteria for GDM (Table 3) (withdrawal statement).

**Table 3:** Prevalence of pregnant women in the ND, MGH, and GDM groups stratified according to diagnostic protocol

	Diagnostic protocols		
	OLD	NEW	*p*
ND	86 (44, 6)	113 (39, 1)	0.2720
MGH	39 (2, 20)	*50 (3, 17)*	0.4928
GDM	68 (35, 2)	126 (43, 6)	0.0818
Total	193	289	482

Results expressed in number (N) and percentage (%)

Test for the comparison of proportions (Chi square)

*ND* non diabetic, *MGH* mild gestational hyperglycemia, *GDM* gestational diabetes mellitus

There were no differences in the prevalence of APNO or in the corresponding relative risk analysis according to the diagnostic protocols tested (Tables 4, 5).

**Table 4:** Perinatal outcomes in the ND, mild gestational hyperglycemia (MGH), and gestational diabetes mellitus (GDM) groups stratified according to diagnostic protocol

	ND (N = 199)			MGH (N = 89)			GDM (N = 194)		
	OLD	NEW	*p*	OLD	NEW	*p*	OLD	NEW	*p*
NB-LGA	0 (0, 0)	9 (8, 0)	*0.0196*	6 (4, 15)	7 (14, 0)	1.0000	6 (8)	14 (1, 11)	0.8006
Macrossomia	3 (4, 4)	5 (4, 4)	1.0000	6 (4, 15)	7 (14, 0)	1.0000	5 (3, 7)	13 (3, 10)	0.6747
First C-section	25 (25, 5)	43 (38, 1)	0.2409	8 (5, 20)	16 (32, 0)	0.3316	16 (5, 23)	30 (8, 23)	1.0000
Length of hospital stay (days)									
1–3	64 (76, 6)	87 (77, 0)	0.8003	29 (74,4)	41 (82, 0)	0.5404	50 (73,5)	96 (76,2)	0.8138
4–7	14 (1, 11)	22 (5, 19)	0.6942	10 (25,6)	8 (16, 0)	0.3911	14 (6, 20)	25 (8, 19)	1.0000
>7	8 (2, 12)	4 (3, 5)	0.1642	0 (0, 0)	1 (2, 0)	1.0000	4 (5, 9)	5 (4, 0)	0.8049
Total	86	113		39	50		68	126	

Results expressed in number (N) and percentage (%)

Test for the comparison of proportions

*NB-LGA* newborn large for the gestational age

**Table 5:** Risk analysis for adverse perinatal outcomes according to diagnostic protocol

	OLD *vs* NEW	
	MGH	GDM
NB-LGA	1.292 (0.473; 3.530)	0.895 (0.275; 2.917)
Macrossomia	0.690 (0.235; 2.025)	1.117 (0.343; 3.683)
First C-section	0.985 (0.492; 1.971)	0.549 (0.206; 1.454)
Δt hospital stay > 7 days	1.172 (0.599; 2.294)	1.521 (0.550; 4.206)

Δt hospital stay length of hospital stay

Results expressed as relative risk (RR) and 95% confidence interval

*NB-LGA* newborn large for the gestational age

## Discussion

In this study, the comparison between the OLD and the NEW GDM diagnostic protocol introduced by the ADA/IADPSG did not reveal any statistically significant differences in the prevalence of GDM and MGH or in the occurrence of APNO at SEDG-FMB/Unesp. The ADA/IADPSG protocol did identify more women with GDM, however, it did not diagnose all cases of MGH as GDM. Of the 289 pregnant women treated under the NEW protocol, 17.3% still maintained the diagnosis of MGH, with normal 75-g OGTT and changed GP.

No statistically significant difference was found in APNO, irrespective of the diagnostic protocol. This finding could be interpreted as negative in relation to the NEW ADA/IADPSG protocol. In this study, a sample of 194 newborns from mothers with GDM, obtained by convenience, may have been insufficient to demonstrate statistically significant results in terms of the occurrence of NB-LGA, macrosomia, first C-section, and longer hospital stays of newborns. A recent Australian study showed beneficial effect of the ADA/IADPSG diagnostic protocol in the occurrence of macrosomia by reducing the risk of developing macrosomia, evaluating 559 pregnant women and newborns [21]. Overall, our results are consistent with the available evidence and show an increase in the prevalence of GDM, ranging from 10 to 25%, and little impact on perinatal outcomes. Further studies are required to define the cost-benefit ratio of these new recommendations [13–17].

Previous results from our research group, show that the cost-effective ratio of maternal and neonatal diagnosis and treatment for prior DM, GDM and MGH is always positive, with social profitability ratio ranging from 1.87 to 5.35 [22]. The treatment protocol was the same for both periods and the 75-g OGTT evaluates two, rather than three, post glucose load plasma glucose levels. Therefore, identifying and treating more women with GDM should not change the cost-benefit indications and the NEW ADA/IADPSG protocol shall be followed at SEDG-FMB/Unesp.

Regarding the association of the GP to the 75-g OGTT for the diagnosis of MGH, it is clear that the new limits and criteria recommended by the ADA/IADPSG also failed to identify 17% of women with MGH, which must be treated to prevent APNO [10]. In this study, all patients with MGH received the same standard treatment as those with GDM and reached average glucose levels and HbA1c within the recommended limits for these pregnancies [20]. This study found 14–15% of NB-LGA and macrosomia, 20–30% of need for a first C-section, and about 10% of newborns with discharge after the third day of life. These pregnant women were already obese in the pre-pregnancy period and despite exhibiting lower levels of fasting glucose and of glucose 2 h after the 75-g OGTT, 17 in 100 evaluated women showed hyperglycemic peaks in GP in response to a normocaloric daily diet [23]. These data support the combined use of the GP and the 75-g OGTT for the diagnosis of GDM and MGH. Indirectly, these results highlight the preventive role of pre-pregnancy obesity control and its metabolic effects in reducing insulin resistance and hyperglycemic disorders in pregnancy, which have a long-term impact on health [7, 8, 10].

This study has some limitations: (1) the study population was a convenience sample, limited to pregnant women who underwent diagnosis tests, prenatal care and delivery at SEDG-FMB/Unesp, to ensure access to all data of interest; (2) the study was a cohort study that has limited applicability for analysis of the association between the diagnostic protocols and perinatal outcomes, (ideally, the protocols would be applied to the same pregnant women, over the same period) and (3) the characteristics of the service itself that, despite being a reference for pregnancies complicated by diabetes, has restricted demand. Nevertheless, our results highlight the validity of the review of each service, enable assessment of local protocols and contribute to the broader discussion and critique of these yet undefined issues.

## Conclusions

The comparison between the OLD and NEW GDM diagnostic protocols showed no statistically significant difference in the prevalence of GDM and MGH or in the occurrence of APNO, at SEDG-FMB/Unesp. However, in absolute numbers, the ADA/IADPSG protocol increased the number of cases diagnosed as GDM by 85.0%, and failed to identify all pregnant women with MGH; the changed GP identified 17.3% of pregnant women living with MGH, despite a normal 75-g OGTT. The results of this study indicate the validity of maintaining GP in the diagnostic protocol of SEDG-FMB/Unesp. Multicenter studies of larger samples should complete the cost-effectiveness of the new GDM diagnostic protocol in preventing APNO.

The use of GP in clinical practice is absolutely feasible because it is an easily performed exam. A negative point could be the adherence of the patients to the examination due to the time of the examination, but in our practice, we observed that patients were once adhered to the screening.

## Abbreviations

GDM: gestational diabetes mellitus
ADA: American Diabetes Association
APNO: adverse perinatal outcomes
MS: metabolic syndrome
DM2: type 2 diabetes mellitus
OGTT: oral glucose tolerance test
GP: glucose profile
SEDG-FMB/Unesp: Specialized Center for Diabetes and Pregnancy of the Clinical Hospital of Botucatu Medical School/Unesp
MGH: mild gestational hyprtglyceia
NB: newborns
LGA: large for gestational age
IADPSG: International *Association of Diabetes and Pregnancy Study Group*
HAPO: hyperglycemia and adverse pregnancy outcome
WHO: world health organization
ND: nondiabetic
NB-LGA: newborn large for gestational age
WG: weight gain
GA: glycemic average

## References

[1] American Diabetes Association (2011). Diagnosis and classification of diabetes mellitus (Position Statement). Diabetes Care.

[2] American Diabetes Association (2012). Diagnosis and classification of diabetes mellitus (Position Statement). Diabetes Care.

[3] American Diabetes Association (2015). Classification and diagnosis of diabetes. Diabetes Care.

[4] Riskin A, Garcia-Prats JA. Up to date: infant of a diabetic mother. In: Weisman LE, Wolfsdorf, JI, Kim MS, editors. http://www.uptodate.com/contents/infant-ofa-diabetic-mother?source=related_link-H714002. Accessed 25 Feb 2016.

[5] Boerschmann H, Pfluger M, Henneberger L, Ziegler AG, Hummel S (2010). Prevalence and predictors of overweight and insulin resistance in offspring of mothers with gestational diabetes mellitus. Diabetes Care.

[6] Vaarasmaki M, Pouta A, Elliot P, Tapanainen P, Sovio U, Ruokonen A (2009). Adolescent manifestations of metabolic syndrome among children born to women with gestational diabetes in a general-population birth cohort. Am J Epidemiol.

[7] Silva MRG, Calderon IMP, Goncalves LC, Aragon FF, Padovani CR, Pimenta WP (2003). Prevalence of diabetes mellitus in women with prior gestational hyperglycemia. Revista de Saúde Pública.

[8] Negrato CA, Jovanovic L, Tambascia MA, Calderon IMP, Geloneze B, Dias A, Rudge MV (2008). Mild gestational hyperglycemia as risk factor for metabolic syndrome in pregnancy and perinatal outcomes. Diabetes Metabol Res Rev.

[9] Rudge MVC, Peracoli JC, Berezowski AT, Calderon IMP, Brasil MAM (1990). The oral glucose tolerance test is a poor predictor of hyperglycemia during pregnancy. Braz J Med Biol Res.

[10] Rudge MV, Calderon IM, Ramos MD, Brasil MAM, Rugolo LMSS, Bossolan G (2005). Hiperglicemia materna diária diagnosticada pelo perfil glicêmico: um problema de saúde pública materno e perinatal. Revista Brasileira de Ginecologia e Obstetrícia.

[11] International Association of Diabetes and Pregnancy Study Groups (IADPSG) (2010). Recommendations on the diagnosis and classification of hyperglycemia in pregnancy (Consensus Panel). Diabetes Care.

[12] HAPO Study Cooperative Research Group (2008). Hyperglycemia and adverse pregnancy outcomes. N Engl J Med.

[13] Falavigna M, Prestes I, Schmidt MI, Duncan BB, Colagiuri S, Roglic G (2013). Impact of gestational diabetes mellitus screening strategies on perinatal outcomes: a simulation study. Diabetes Res Clin Pract.

[14] Holt RIG, Coleman MA, McCance DR (2011). The implications of the new International Association of Diabetes and Pregnancy Study Groups (IADPSG) diagnostic criteria for gestational diabetes. Diabet Med.

[15] Leary J, Pettitt DJ, Jovanovic L (2010). Gestational diabetes guidelines in a HAPO world. Best Pract Res Clin Endocrinol Metabol.

[16] Visser GA, de Valk HW (2013). Is evidence strong enough to change diagnostic GDM criteria?. Am J Obstet Gynecol.

[17] Mission JF, Ohno MS, Cheng YW, Caughey AB (2012). Gestational diabetes screening with the new IADPSG guidelines: a cost-effectiveness analysis. Am J Obstet Gynecol.

[18] World Health Organization (2014). Diagnostic criteria and classification of hyperglycaemia first detected in pregnancy. Diabetes Res Clin Pract.

[19] American Diabetes Association (2010). Diagnosis and classification of diabetes mellitus (Position Statement). Diabetes Care.

[20] Metzger BE, Buchanan TA, Coustan DR (2007). Summary and recommendations of the Fifth International Workshop-Conference on Gestational Diabetes Mellitus. Diabetes Care.

[21] Laafira A, White SW, Griffin CJ, Graham D. Impact of the new IADPSG gestational diabetes diagnostic criteria on pregnancy outcomes in Western Australia. Aust NZ J Obstet Gynaecol. 2015. doi: 10.1111/ajo.12394 (Epub ahead of print). 10.1111/ajo.12394 26293845

[22] Molina Cavassini AC, Molina Lima SA, Calderon IMP, Rudge MVC (2012). Cost-benefit of hospitalization compared with outpatient care for pregnant women with pregestational and gestational diabetes or with mild hyperglycemia, in Brazil. Sao Paulo Med J.

[23] American Dietetic Association (2008). Position of the American Dietetic Association: nutrition and Lifestyle for a Healthy Pregnancy Outcome. J Am Diet Assoc.

